# Indole-3-acetic acid (IAA) protects *Azospirillum brasilense* from indole-induced stress

**DOI:** 10.1128/aem.02384-24

**Published:** 2025-03-25

**Authors:** Elena E. Ganusova, Ishita Banerjee, Trey Seats, Gladys Alexandre

**Affiliations:** 1 Biochemistry and Cellular and Molecular Biology Department, University of Tennessee 196197 , Knoxville, Tennessee, USA; Georgia Institute of Technology, Atlanta, Georgia, USA

**Keywords:** *Azospirillum*, auxin, indoles, stress responses, plant root colonization

## Abstract

*Azospirillum brasilense* is plant-growth promoting rhizobacteria that produces the phytohormone indole-3-acetic acid (IAA) to induce changes in plant root architecture. The major pathway for IAA biosynthesis in *A. brasilense* converts tryptophan into indole-3-pyruvic acid (I3P) and then, through the rate-limiting enzyme, indole-3-pyruvate decarboxylase (IpdC), into IAA. Here, we characterize the potential role for IAA biosynthesis in the physiology of these bacteria by characterizing the expression pattern of the *ipdC* promoter, analyzing an *A. brasilense ipdC* mutant using multiple physiological assays and characterizing the effect of I3P, which likely accumulates in the absence of *ipdC* and affects bacterial physiology. We found that the *ipdC* mutant derivative has a reduced growth rate and an altered physiology, including reduced translation activity as well as a more depolarized membrane potential compared to the parent strain. Similar effects could be recapitulated in the parent strain by exposing these cells to increasing concentrations of I3P, as well as other indole intermediates of IAA biosynthesis. Our results also indicate a protective role for IAA against the harmful effects of indole derivatives, with exogenous IAA restoring the membrane potential of cells exposed to indole derivatives for prolonged periods. These protective effects appeared to restore cell physiology, including in the wheat rhizosphere. Together, our data suggest that the IAA biosynthesis pathway plays a major role in *A. brasilense* physiology by maintaining membrane potential homeostasis and regulating translation, likely to mitigate the potential membrane-damaging effects of indoles that accumulate during growth under stressful conditions.

**IMPORTANCE:**

IAA is widely synthesized in bacteria, particularly in soil and rhizosphere bacteria, where it functions as a phytohormone to modulate plant root architecture. IAA as a secondary metabolite has been shown to serve as a signaling molecule in several bacterial species, but the role of IAA biosynthesis in the physiology of the producing bacterium remains seldom explored. Results obtained here suggest that IAA serves to protect *A. brasilense* from the toxic effect of indoles, including metabolite biosynthetic precursors of IAA, on membrane potential homeostasis. Given the widespread production of IAA in soil bacteria, this protective effect of IAA may be conserved in diverse soil bacteria.

## INTRODUCTION

Indoles are compounds derived from tryptophan (Trp) produced by various bacteria, including *Escherichia coli*, *Proteus vulgaris*, *Burkholderia unamae*, *Vibrio cholerae*, *Pseudomonas syringae*, different species of *Xanthomonas*, and *Klebsiella*, and can act as signaling molecules (1–9). In *E. coli*, the most studied indole-producing bacteria, indole biosynthesis increases during the stationary phase (10). Indole is freely diffusible across bacterial membranes (11), and in millimolar concentrations, indole can act as a proton ionophore, resulting in inhibition of cell division (12). Indole produces opposite effects on modulating diverse physiological and metabolic functions in bacteria, including ion transport (8), cytoplasmic pH homeostasis (13, 14), flagellar motor activity (15), chemotaxis behavior (16), resistance to antibiotics (17), and other stress responses (18–20). Indoles have been implicated in interbacterial species communication (6, 21–23) and virulence (24, 25). Depending on concentration and the species considered, indoles have been described as having opposing effects on persister cells and biofilm formation (6, 14, 16, 26). Several of the effects of indole on cell physiology are consistent with indoles altering membrane integrity and membrane potential (27, 28). Other studies suggest that indoles may inhibit DNA gyrase *in vitro* (29), promote ribosome hibernation (30), or target membrane tension or protein activities (14). One possibility that seems plausible, given the diverse effects observed, is that indoles have a range of affinity for multiple cellular and molecular targets and thus could have pleiotropic effects on bacterial physiology.

Indole‐3‐acetic acid (IAA) is an indole-derived compound produced by bacteria, plants, and fungi (31, 32). In plants, IAA is a family of indole-derived compound derivatives produced by plants and referred to as auxin (32). Auxin acts as a phytohormone regulating multiple aspects of plant growth and development (32). IAA is also widely synthesized in bacteria, particularly in soil and rhizosphere bacteria, with up to 80% of them producing IAA (33, 34). Several studies have shown that IAA is a signaling molecule in bacteria mediating changes in gene expression in multiple plant-associated bacterial species. Genes regulated by IAA include stress resistance (35), antibiotic synthesis (36, 37), virulence (7), and type VI secretion system (38). While IAA appears to alter the transcriptomes of bacterial species, specific bacterial receptors that directly bind IAA have been identified only in a few bacterial species, including *Serratia plymuthica* (39) and *Pseudomonas putida* (40). These observations collectively support the hypothesis that IAA serves as a signaling molecule in bacteria.

The α-proteobacterium *Azospirillum brasilense* is a plant growth-promoting bacterium (41). Multiple mechanisms have been invoked to explain the plant growth-promoting effect of *A. brasilense*, including nitrogen fixation and phytohormone production, such as auxin production (42, 43). *A. brasilense* cells synthesize several indole derivatives, including IAA. IAA biosynthesis was suggested to occur through several different pathways in this species (44, 45). The majority (90%) of IAA produced by azospirilla is produced via the tryptophan-dependent pathway, with the indole-3-pyruvate decarboxylase (IpdC) being the rate-limiting enzyme (44). Multiple studies in diverse strains of *Azospirillum* showed that knocking out *ipdC* abolishes about 90% of IAA biosynthesis (44, 45). Another 10% of IAA was initially hypothesized to be synthesized through a tryptophan-independent pathway (45), but later studies questioned this possibility (46). The origin of the remaining 10% of IAA produced in the absence of IpdC remains unknown. In *A. brasilense*, IAA biosynthesis (and maximal induction of the *ipdC* promoter) occurs in the stationary phase of growth, similar to what has been described in other bacterial species (47–49). Conditions that reduce the growth rate also increase IAA biosynthesis and *ipdC* promoter activity in *A. brasilense* (49). *A. brasilense* does not catabolize or grow on IAA (50). While IAA was proposed to modulate transcriptional changes in this species (51), no specific influx/efflux transporters for IAA or any transcription factor binding IAA has been identified yet. While the role of IAA in the interaction of *A. brasilense* with plant roots is well described (31, 52, 53), the role that IAA plays in the physiology of these bacteria is not clear. In this study, we characterize the potential role for IAA biosynthesis in the physiology of *A. brasilense* by characterizing the expression pattern of the *ipdC* promoter and analyzing an *A. brasilense ipdC* mutant using multiple physiological assays. Results obtained suggest that IAA serves to protect *A. brasilense* from the toxic effect of indoles on membrane potential homeostasis. Given the widespread production of IAA in soil bacteria and the distribution of the IpdC-dependent pathway in bacteria, we hypothesize this protective effect of IAA is likely conserved in diverse soil bacteria.

## RESULTS

### Expression pattern of the *ipdC* promoter and IAA production

**Fig 1:**
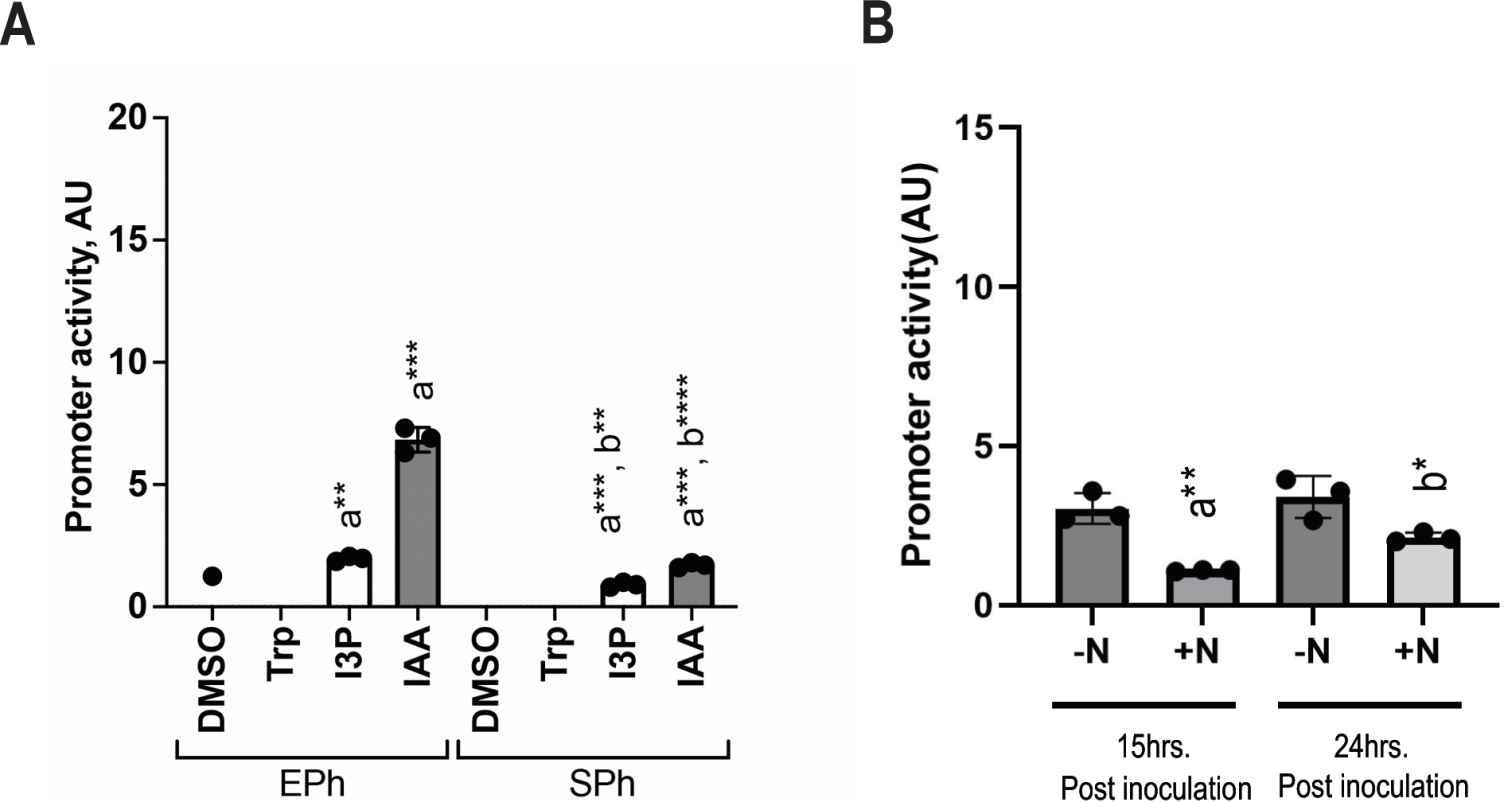
Promoter activity of the *A. brasilense ipdC* gene. (**A**) Promoter activity of *ipdC* in wild-type (WT) *A. brasilense* cells in the exponential phase (Eph) and stationary phase (SPh) in the presence of 100 µM of tryptophan (Trp), I3P, and IAA. DMSO was used as a negative control. Error bars represent the standard deviation. a, significantly different from DMSO; b, significantly different from the exponential phase. *P = 0.1, **P = 0.01, ***P = 0.001, ****P = 0.0001 (by Student’s *t*-test). (**B**) Expression pattern of the *ipdC* promoter in *A. brasilense* wild type (Sp7) in minimal medium for *Azospirillum brasilense* under conditions of nitrogen fixation (−N) and in the presence of ammonium (+N) 15 and 24 h post-inoculation. Values indicating promoter activity were normalized to the values obtained from cultures containing an empty vector. Error bars represent the standard deviation. *P = 0.1, **P = 0.01 (by Student’s *t*-test). a, significant in comparison with –N condition at 15 h post-inoculation; b, in comparison with –N condition at 24 h post-inoculation.

To evaluate the role of IAA production in *A. brasilense* physiology, we first analyzed the pattern of expression of *ipdC* during the exponential and stationary phase of growth in cells grown in a minimal medium with ammonium chloride. Under these growth conditions, we also tested the effect of tryptophan (negative control) and IAA (positive control), along with a precursor of IAA in the IpdC-dependent pathway from tryptophan, namely, indole-3-pyruvic acid (I3P) (Fig. 1A). Consistent with published studies (45), the addition of IAA increased the *ipdC* promoter activity (P*_ipdC_*), while the addition of tryptophan did not have any effect (Fig. 1A). The addition of I3P also significantly increased P*_ipdC_* activity. The effects of I3P and IAA on the P*_ipdC_* activity were greater in exponentially growing cells than in stationary phase cells. Next, we compared the effects of growth under nitrogen-fixation conditions (no combined nitrogen source and no aeration of cultures) to growth with ammonium chloride (and with aeration) on the P*_ipdC_* activity. Because cells do not significantly grow under conditions of nitrogen fixation, we first grew them in the presence of ammonium to mid-log phase, washed the cell pellet, and then induced cells in a nitrogen-free minimal medium for 15 or 24 h, without shaking (Fig. 1B). The comparison indicated that P*_ipdC_* activity is greater under nitrogen-fixation conditions regardless of incubation times and that it increases over time in the presence of ammonium chloride. Together, the data indicate that a precursor of IAA, I3P, as well as IAA significantly induce P*_ipdC_* and that conditions of reduced or persistent lack of a combined source of nitrogen (nitrogen-fixation conditions or extended growth in the presence of ammonium chloride) also induce the promoter for *ipdC*. The induction of *ipdC* is thus tightly controlled with nitrogen metabolism and a precursor of IAA biosynthesis, I3P.

### An *ipdC* knockout results in a growth defect and changes in the cell morphology of *A. brasilense*

**Fig 2:**
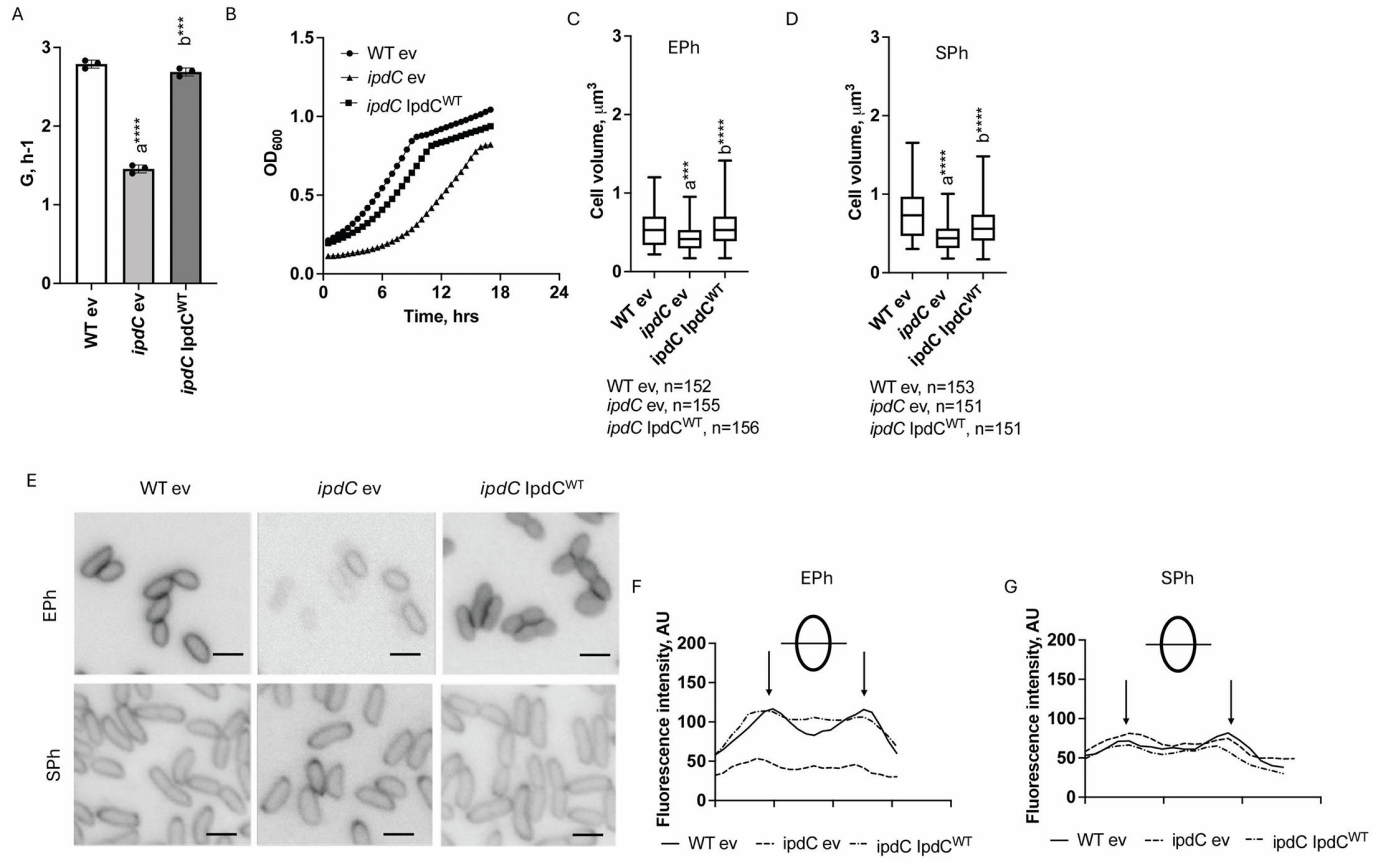
*ipdC* knockout affects cell physiology of *A. brasilense*. (**A**) Growth rate of WT ev, *ipdC* ev, and *ipdC* IpdC^WT^ strains in MMAB. Error bars represent the standard deviation. Error bars represent the standard deviation. a, significantly different from WT ev; b, significantly different from *ipdC* ev. ***P = 0.001, ****P = 0.0001 (by Student’s *t*-test). (**B**) Representative growth curves of WT ev, *ipdC* ev, and *ipdC* IpdC^WT^ strains in MMAB. G, h^−1^ is the growth rate. (**C**) Average cell volume of WT ev, *ipdC* ev, and *ipdC* IpdC^WT^ cells grown to the exponential phase (Eph) in MMAB. Error bars represent the standard deviation. a, significantly different from WT ev; b, significantly different from *ipdC* ev. *P = 0.1, ****P = 0.0001 (by Student’s *t*-test). (**D**) Average cell volume of WT ev, *ipdC* ev, and *ipdC* IpdC^WT^ cells grown to the stationary phase (SPh) in MMAB. Error bars represent the standard deviation. a, significantly different from WT ev; b, significantly different from *ipdC* ev. ***P = 0.001, ****P = 0.0001 (by Student’s *t*-test). (**E**) Absence of *ipdC* affects cell-impermeant dye (1 µg/mL of FM4-64) membrane staining of *A. brasilense* cells. Representative fluorescent microscopy images of WT ev, *ipdC* ev, and *ipdC* IpdC^WT^ cells grown in MMAB to Eph or SPh in MMAB. The scale bar is 2 µm. Fluorescent profile of the WT ev, *ipdC* ev, and *ipdC* IpdC cells grown in MMAB to the Eph (**F**) or to the SPh (**G**).

Next, we constructed an *A. brasilense ipdC::Gm^r^* insertion mutant (later referred to as *ipdC*) to characterize the role of IAA biosynthesis in bacterial physiology. Mutant derivatives of *Azospirillum* spp. lacking *ipdC* have been previously constructed and characterized for IAA production and plant growth promotion, with experiments regarding growth rates typically conducted in rich complex media (45, 54–56). Here, we compared the growth of the wild-type (WT) strain Sp7 and the *ipdC* mutant derivative in a minimal medium supplemented with ammonium chloride as a source of nitrogen (Fig. 2A and B). We used WT and *ipdC* strains carrying an empty vector control (WT ev and *ipdC* ev, respectively), as well as an *ipdC* mutant strain expressing the parental *ipdC* from a plasmid (*ipdC* IpdC^WT^). The WT ev strain growth rates were 2.19 ± 0.05 h⁻¹ in minimal medium for *Azospirillum brasilense* (MMAB). In comparison, the *ipdC* ev strain growth rate was 1.46 ± 0.05 h^−1^. The growth rate of the *ipdC* IpdC^WT^ was similar to the WT ev, 2.3 ± 0.7 h⁻¹ (Fig. 2A). The expression of the parental *ipdC* gene on a low-copy vector thus restored growth defects caused by inactivation of *ipdC* (Fig. 2A and B). The reduced growth rate of the *ipdC* ev strain was also characterized by increased duration of the latent phase (about 6 h) and a delayed entry into the stationary phase compared to the WT ev and *ipdC* IpdC^WT^ (Fig. 2B). We stained the cells’ membranes with FM4-64 dye to measure the volume of the WT ev, *ipdC* ev, and *ipdC* IpdC^WT^ cells grown to exponential and stationary phases in MMAB (Fig. 2C and D). The cell volume of the *ipdC* ev strain was significantly lower than in WT ev or *ipdC* IpdC^WT^ strains in the exponential or stationary phase of growth (Fig. 2C and D). These observations are consistent with a growth impairment caused by the disruption of *ipdC*. We also observed that cell membranes of the *ipdC* ev strain grown to the mid-exponential phase did not stain as well as WT ev and *ipdC* IpdC^WT^ membranes with the FM4-64 dye, for cells grown to the exponential phase (Fig. 2E and F). Differences were not observed in the stationary phase of growth (Fig. 2E and G). Cells of the *ipdC* mutant thus have a growth defect that affects cellular morphology (volume) and physiology (FM 4–64 staining).

**Fig 3:**
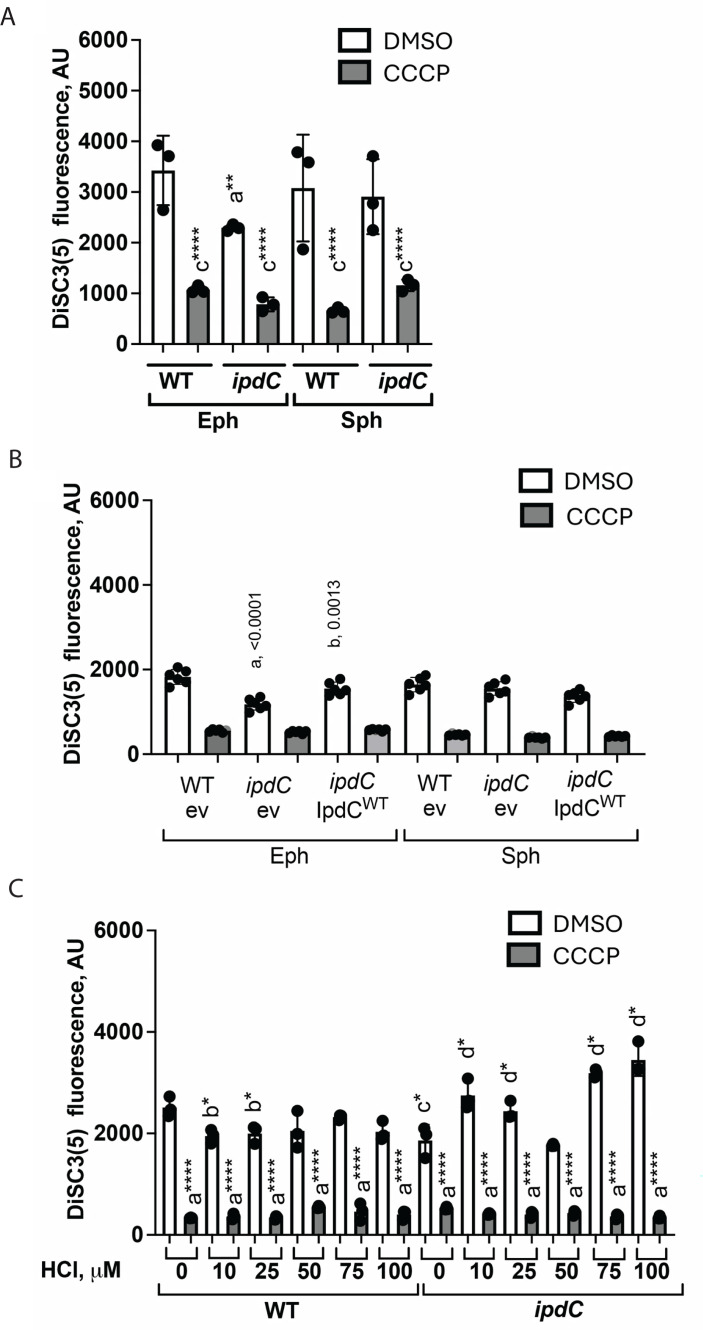
Resting membrane potential is affected in *ipdC* cells. (**A**) Resting (with DMSO) and collapsed (with CCCP) membrane potential of WT and the *ipdC* strains grown to exponential phase (Eph) or stationary phase (Sph) measured using 1 µM of DisC3(5) fluorescent probe. DMSO treatments represent the resting membrane potential of cells. Error bars represent the standard deviation. a, significantly different from DMSO; b, significantly different from WT. **P = 0.01, ****P = 0.0001 (by Student’s *t*-test). (**B**) Functional complementation of the *ipdC* resting membrane potential (with DMSO) and collapsed (with CCCP). WT ev, *ipdC* ev, and *ipdC* IpdC^WT^ cells were grown to Eph or Sph, and membrane potential was measured using 1 µM of DisC3(5) fluorescent probe. Error bars represent the standard deviation (*P* values are shown above the graphs indicating resting membrane potential [by Student’s *t*-test]). (**C**) Resting (with DMSO) and collapsed (with CCCP) membrane potential of WT and the *ipdC* strains grown to the exponential phase and exposed to 0–100 μM of HCl. Error bars represent the standard deviation. a, significantly different from DMSO; b, significantly different from control (0 µM of HCl); c, significantly different from WT; d, significantly different from *ipdC* with DMSO. *P = 0.1, ****P = 0.0001 (by Student’s *t*-test).

To test whether the growth defect and altered physiology of the *ipdC* mutant strain were due to disrupted membrane energetics of *A. brasilense*, we used a fluorescent, potentiometric cationic reporter, DiSC3(5), to assess the membrane potential (Δψ). DiSC3(5) fluoresces when accumulated within polarized cells, and fluorescence decreases upon membrane depolarization (57). First, we compared the resting membrane potential of cells from the WT and *ipdC* mutant strains at different growth stages. As expected from the growth rate defect, the resting membrane potential of *ipdC* cells during exponential growth was significantly lower compared to WT cells but did not differ in the stationary phase (Fig. 3A). As a control for DiSC3(5) fluorescence reporting on the membrane potential, we used carbonyl cyanide *m*-chlorophenylhydrazone (CCCP) to depolarize the cells’ membrane potential, and, as expected, the addition of CCCP caused DiSC3(5) fluorescence to decrease relative to the dimethyl sulfoxide (DMSO) controls in all tested strains (Fig. 3A). The membrane potential defect of the strain lacking *ipdC* in the exponential phase of growth could be complemented by expressing the parental *ipdC* gene *in trans* and had no effect in the stationary phase of growth (Fig. 3B).

We hypothesized that the reduced membrane potential of the strain lacking *ipdC* should translate into disrupted membrane potential homeostasis. We also surmise that an altered membrane potential homeostasis should become apparent by challenging the cells with stressors that perturb the membrane potential. To test this hypothesis, we exposed cells of the WT and *ipdC* mutant strains to equimolar concentrations of HCl (10–100 μM), which can disrupt the membrane electrical balance by altering proton concentration gradients across the membrane (Fig. 3C). The resting membrane potential of WT cells remained unaffected by exposure to increased concentration of HCl, consistent with a robust membrane homeostasis in this strain (Fig. 3C). In contrast, the resting potential of the *ipdC* mutant increased in the presence of 10, 25, 75, and 100 µM of HCl, though it was not measurably affected at 50 µM HCl (Fig. 3C). We do not exactly know why the 50 µM HCl concentration did not produce a measurable membrane potential change in the mutant strain. Some possibilities include a unique combined effect of HCl on protons and charge gradient across the cell membranes at this concentration that abolish major drivers of the changes in membrane potential under these conditions. This result suggests that the strain lacking *ipdC* is unable to maintain membrane potential homeostasis. Treatment with 1 µM of the CCCP ionophore led to a significant reduction in DiSC3(5) fluorescence in comparison with DMSO controls, consistent with the fluorescence changes reporting on changes in membrane potential. The data collectively support the hypothesis that the *ipdC* mutant has a growth defect that is likely due to its inability to maintain membrane potential homeostasis.

### Indole-3-pyruvate causes membrane depolarization of *A. brasilense* cells

**Fig 4:**
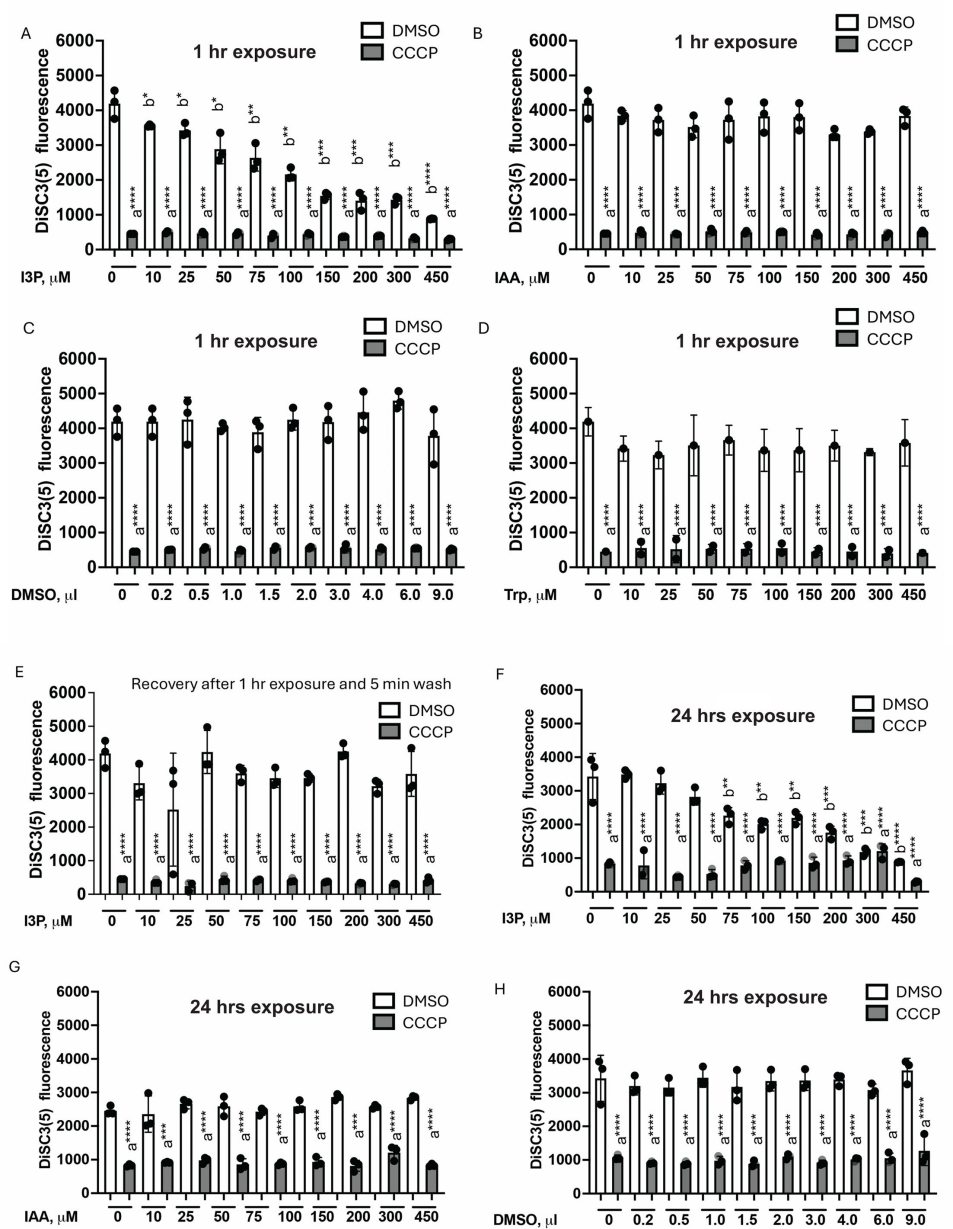
Membrane potential of WT *A. brasilense* is negatively affected by I3P but not by IAA. Resting (with DMSO) and collapsed (with CCCP) membrane potential of WT cells grown to mid-exponential phase and exposed for 1 h to 0–450 μM of I3P (**A**), to 0–450 μM of IAA (**B**), an equal volume of DMSO as a solvent (**C**), and to 0–450 μM of tryptophan (Trp) (**D**). Error bars represent the standard deviation. Error bars represent the standard deviation. a, significantly different from DMSO; b, significantly different from control (0 µM). *P = 0.1, **P = 0.01, ***P = 0.001, ****P = 0.0001 (by Student’s *t*-test). (**E**) Resting (with DMSO) and collapsed (with CCCP) membrane potential of WT cells grown to mid-exponential phase and exposed for 1 h to 0–450 μM of I3P, washed for 5 min, and re-suspended in MMAB without I3P. Error bars represent the standard deviation. a, significantly different from DMSO. ****P = 0.0001 (by Student’s *t*-test). (**F**) Resting (with DMSO) and collapsed (with CCCP) membrane potential of WT cells grown to mid-exponential phase and exposed for 24 h to 0–450 μM of I3P. Error bars represent the standard deviation. a, significantly different from DMSO. **P = 0.01, ***P = 0.001, ****P = 0.0001 (by Student’s *t*-test). (**G**) Resting (with DMSO) and collapsed (with CCCP) membrane potential of WT cells grown to mid-exponential phase and exposed for 24 h to 0–450 μM of IAA. Error bars represent the standard deviation. a, significantly different from DMSO. ****P = 0.0001 (by Student’s *t*-test). (**H**) Resting (with DMSO) and collapsed (with CCCP) membrane potential of WT cells grown to mid-exponential phase and exposed for 24 h to various volumes of DMSO. Error bars represent the standard deviation. a, significantly different from DMSO. ****P = 0.0001 (by Student’s *t*-test).

Why would the *ipdC* mutant have a reduced membrane potential and compromised membrane homeostasis? The IpdC-dependent pathway for IAA biosynthesis represents ~90% of the total IAA produced by *A. brasilense* (45). An *ipdC* mutant strain could be expected to accumulate indole precursor(s) of IAA such as indole-3-pyruvate (I3P) since it cannot be converted to IAA. At high concentrations, indoles are proton ionophores that depolarize cell membranes (12). We hypothesized that the accumulation of I3P in the *ipd*C mutant could contribute to lowering the membrane potential. We tested the effect of I3P, IAA, and tryptophan on the membrane potential by exposing cells to increasing concentrations of these compounds for 1 h (Fig. 4A through D). We found that while the exogenous addition of I3P depolarized the membrane of *A. brasilense* WT in a concentration-dependent manner, neither IAA nor tryptophan, the precursor of both IAA and I3P produced this effect, and these compounds mirrored the effect of DMSO (Fig. 4A through D). The effect of I3P on the membrane potential was abolished when cells were washed following a 1 h exposure to different concentrations of I3P (Fig. 4E), suggesting that the effect of I3P on membrane potential is quickly reversible. Similar effects on the membrane potential were observed under persistent I3P exposure (24 h) (Fig. 4F), while IAA or the solvent control DMSO did not produce this effect (Fig. 4G and H).

### IAA can protect cells from the membrane potential disrupting effect of indol-3-pyruvate

**Fig 5:**
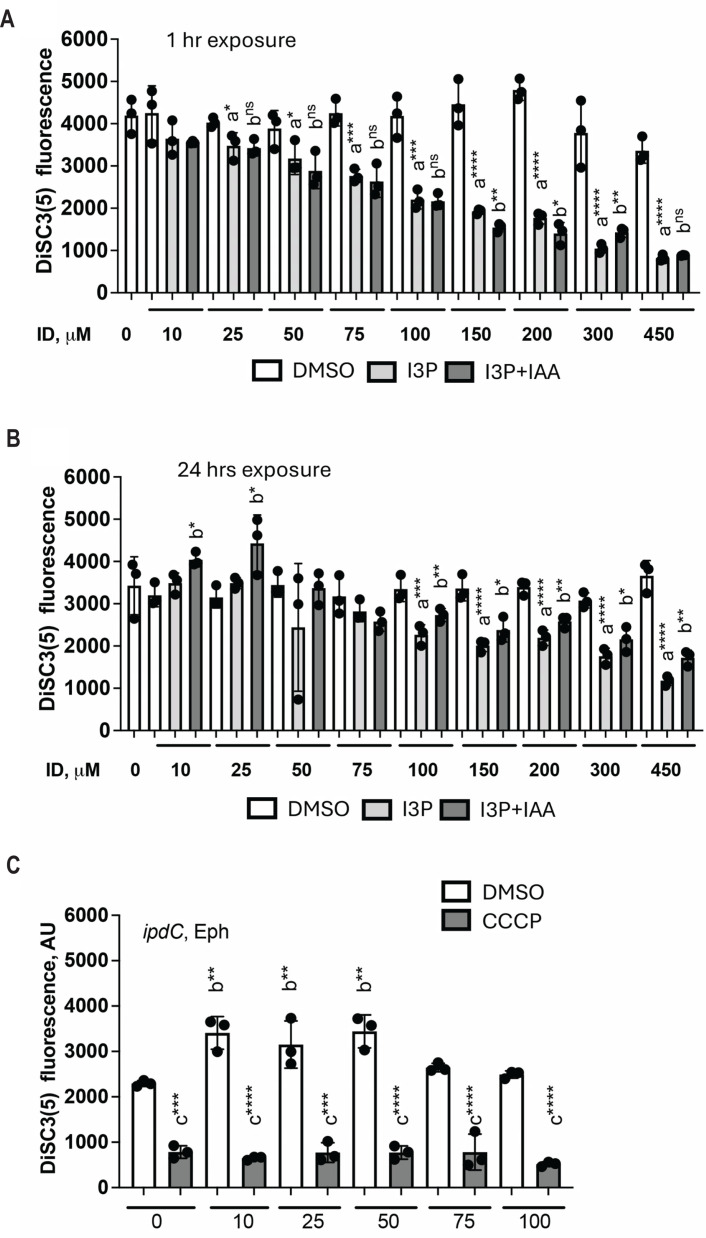
Resting (with DMSO) membrane potential of WT cells grown to the exponential phase and exposed to 0–450 μM of I3P or to 0–450 μM of I3P with addition of 100 µM of IAA or DMSO (solvent control) to 1 h (**A**) or 24 h (**B**). Error bars represent the standard deviation. a, significantly different from control (0 µM); b, significant in comparison with control (0 µM); ns, not significant (by Student’s *t*-test). *P = 0.1, **P = 0.01, ***P = 0.001, ****P = 0.0001. (**C**) Resting (with DMSO) and collapsed (with CCCP) membrane potential of *ipdC* cells exposed to 0–100 µM of IAA. Error bars represent the standard deviation. a, significantly different from DMSO; b, significantly different from control (0 µM); ns, not significant (by Student’s *t*-test). **P = 0.01, ***P = 0.001, ****P = 0.0001.

Because IAA does not have any measurable impact on the membrane potential, but one of its precursor molecules, I3P, does, we hypothesized that IAA could protect cells against the toxic effects of I3P. We tested this possibility by measuring the membrane potential of cells treated with different concentrations of I3P with and without the addition of 100 µM IAA for 1 h (Fig. 5A) or for 24 h (Fig. 5B). Under conditions of acute exposure to I3P, the membrane potential of the cells treated with I3P or with I3P and IAA were similar and lower than that without I3P, indicating that IAA does not protect cells against the membrane potential-damaging effect of I3P when both are added exogenously for a short time. Contrasting results were observed when cells were incubated for 24 h (Fig. 5B). At low concentrations of I3P (10–25 μM), the addition of IAA promoted an elevated membrane potential relative to the resting membrane potential of cells under control conditions (Fig. 5B). There was no difference in the resting membrane potential of cells treated with intermediate concentrations of I3P (50–75 μM) regardless of the addition of IAA, suggesting that the cells may adapt to the presence of I3P, regardless of whether IAA is added. At elevated concentrations of I3P (>100 µM), IAA partially mitigated the effect of I3P on the membrane potential (Fig. 5B). To further test the hypothesis of the protecting role of IAA, we compared the impact of increasing exogenous concentrations of IAA for 1 h on the membrane potential of the *ipdC* mutant (Fig. 5C). During the exponential phase of growth, smaller concentrations of IAA (10–50 μM) restored the membrane potential of the *ipdC* mutant strain to WT levels, while higher concentrations (75–100 μM) did not (Fig. 5D). Therefore, IAA may protect the cells in two different ways: in cells with lower membrane potential, IAA helps cells restore a membrane potential, and IAA also mediates an adaptation of cells to lower concentrations of a precursor of IAA, I3P, when incubated for prolonged periods with IAA (24 h).

### Other indole derivatives have transient effects on the resting membrane potential of *A. brasilense*

**Fig 6:**
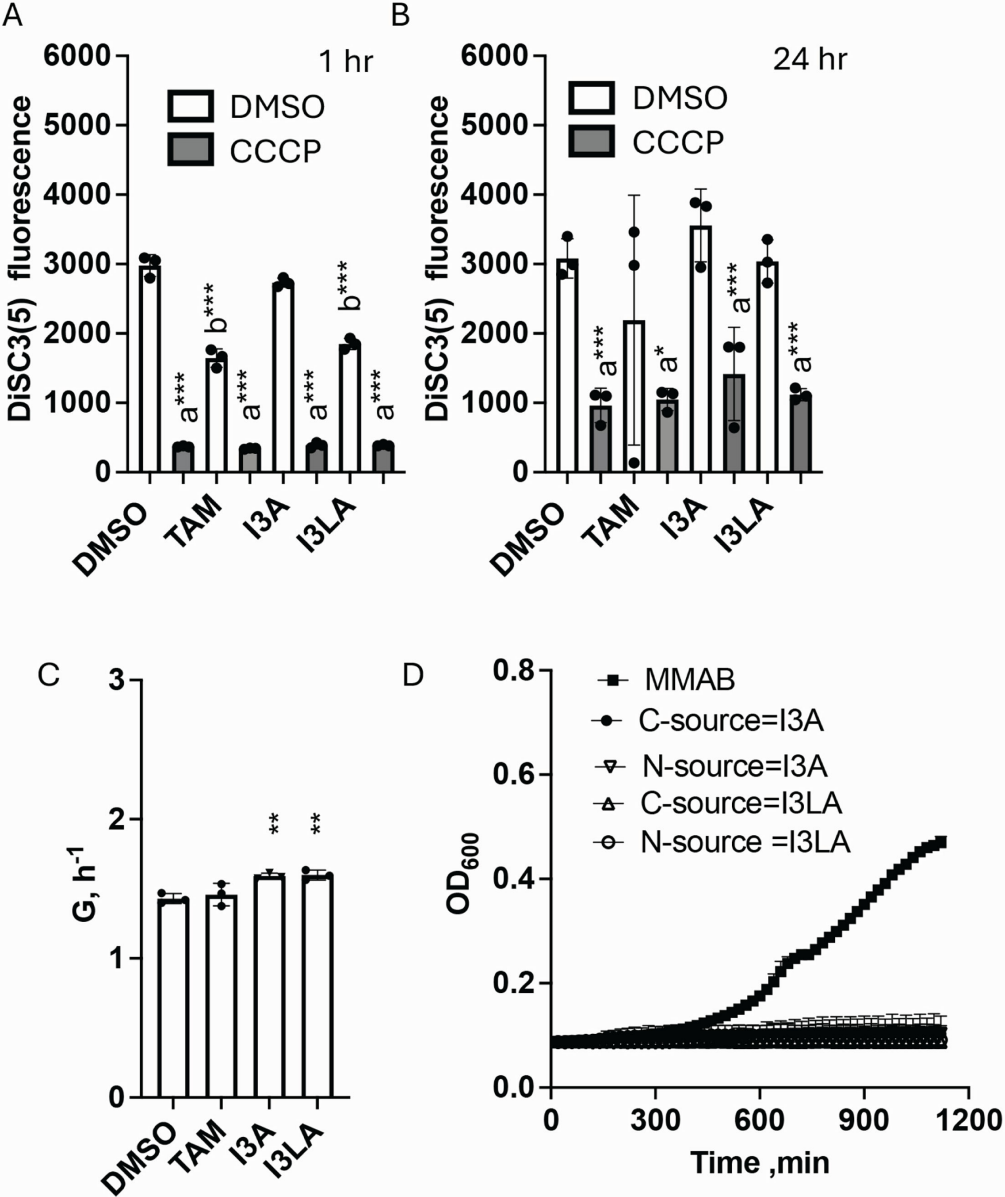
Characterization of the membrane potential of WT *A. brasilense* in the presence of 100 µM of indole derivatives tryptamine (TAM), indole-3-acetamide (I3A), or indole-3-lactic acid (I3LA). Resting (with DMSO) and collapsed (with CCCP) membrane potential of WT cells grown to mid-exponential phase and exposed to 100 µM of TAM, I3A, I3LA, or DMSO (control) for 1 h (**A**) or 24 h (**B**). Error bars represent the standard deviation. a, significantly different from DMSO; b, significantly different from control (0 µM). *P = 0.1, **P = 0.01, ***P = 0.001 (by Student’s *t*-test. (**C**) Growth rate of WT cells grown in MMAB with 100 µM of TAM, I3A, or I3LA. G, h^−1^ is the growth rate. Error bars represent the standard deviation (significantly different from control). **P = 0.01 (by Student’s *t*-test). (D) Growth of WT cells in the presence of 100 µM I3A or I3LA as a carbon or nitrogen source.

Previous studies reported that *A. brasilense* cells synthesize several indole derivatives from tryptophan—tryptamine (TAM), indole-3-acetamide (I3A), and indole-3-lactic acid (I3LA)—although pathways involved are not described (46, 58, 59). When exposed to 100 µM of TAM or 100 µM I3LA for 1 h, the resting membrane potential of the WT cells decreased significantly but not that of cells treated with I3A (Fig. 6A). Prolonged exposure (24 h) to 100 µM TAM, I3A, or I3LA did not affect the membrane potential of the bacterial cells (Fig. 6B). TAM did not affect the growth rate of the WT cells, although I3A and I3LA slightly increased the growth rate of *A. brasilense* (Fig. 6C). Neither I3A nor I3LA could be used as a sole nitrogen or carbon source by *A. brasilense* (Fig. 6D). We do not know whether the effect of I3A is related to this compound added exogenously and not being transported or if the concentration used here is too low to see an effect. While we tested some indoles that are some of the precursors of IAA in this study, we do know the diversity of indoles that the cells studied here may produce. Therefore, we used the Salkowski reagent, which has broad specificity for indoles including IAA as well as many other indole derivatives (60) to compare indoles detected in the cell pellets, in the extracellular medium or in the combined compartments in WT and *ipdC* mutant strains grown in minimal medium with or without tryptophan (Fig. S1). We only detected significant indoles in the intracellular compartment and the extracellular medium of cells grown with tryptophan, but there were no significant differences between the WT and the *ipdC* mutant strains. This indicates that the difference between the strains is likely in the type of indoles they produce, since the *ipdC* strain is not expected to produce much IAA. Together, the data indicate that both cell types produce indoles and that indoles produced from tryptophan catabolism in *A. brasilense* (e.g., I3LA and I3P) can reduce the membrane potential.

### ipdC cells may have reduced translation

**Fig 7:**
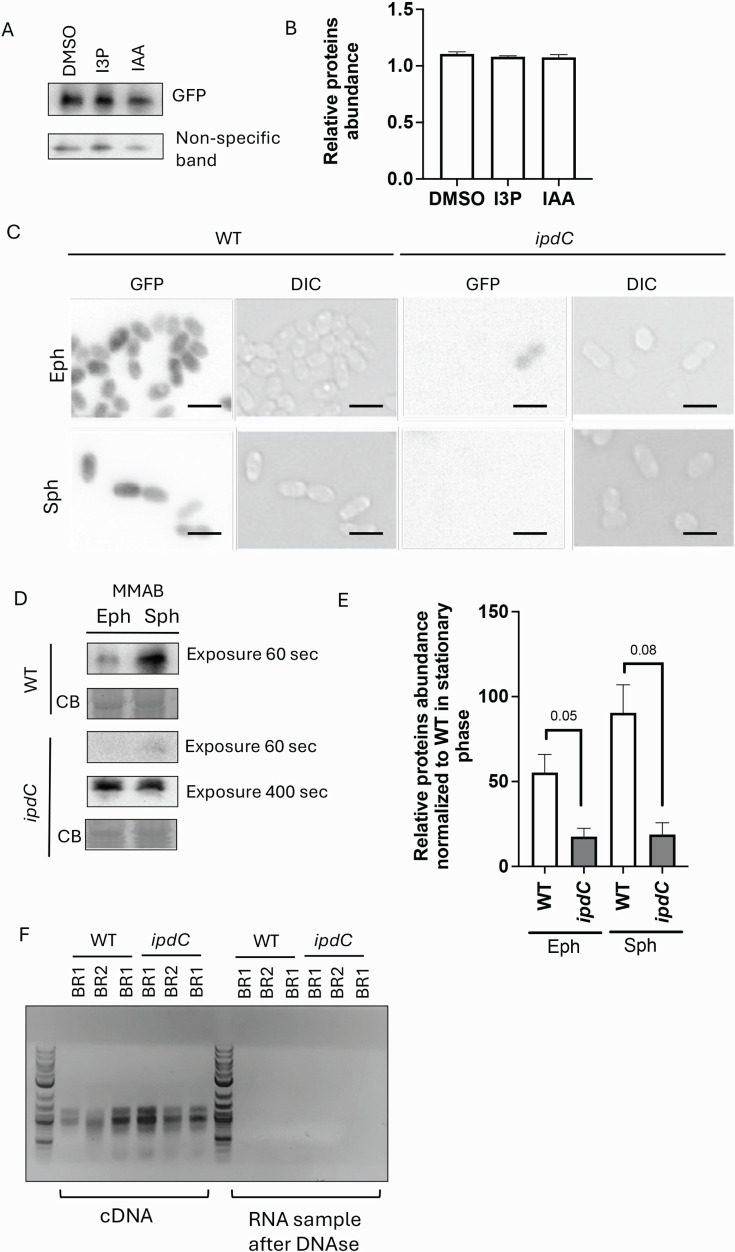
Translation of GFP (model protein) is not affected by I3P in WT cells but affected upon deletion of *ipdC* in *A. brasilense*. (**A**) GFP amount is not affected in the WT cell growth to the exponential phase and exposed to DMSO, 100 µM I3P, or 100 µM IAA for 24 h. A representative Western blot is shown. Non-specific band detected by Western blot was used as a loading control. (**B**) Quantitation of the GFP bands from panel A. (**C**) Representative fluorescent microscopy images of WT and *ipdC* cells expressing GFP under constitutive promoter and grown in MMAB to exponential phase (EPh) or stationary phase (SPh). Fluorescent images were collected at 2 seconds of exposure. Scale bar is 2 µm. (**D**) Representative Western blots of WT and *ipdC* cells expressing GFP. Cells were collected in the Eph and SPh phases. Chemiluminescence signal collected after 60 seconds of exposure was barely detectable in the *ipdC* case; longer exposure (400 seconds) produced a clearly visible band. (**E**) Relative protein abundance of GFP in WT and ipdC cells (quantitation was done using blots exposed for 60 seconds). Band quantification was done using ImageJ. CB, Coomassie blue staining. Error bars represent the standard deviation. The *P* value indicates a significant difference between WT and *ipdC* cells in the Student *t*-test. (**F**) *gfp* transcription is not affected in *ipdC* cells. The left panel represents gel electrophoresis images of three biological replicas of cDNA-based PCR of WT and *ipdC* cells expressing *gfp* (left panel) and controls after DNase treatment of RNA samples.

It was previously shown that indole can cause protein misfolding in bacterial cells (61). We first tested green fluorescent protein (GFP) expression in WT cells treated with I3P or IAA for 24 h using Western blots in cells carrying a low-copy vector expressing *gfp* from a constitutive promoter (Fig. 7A). We did not detect any change in the abundance of GFP regardless of treatment conditions (Fig. 7A and B). We next compared GFP expression in cells of the WT and the *ipdC* mutant strains (Fig. 7C). In both exponential and stationary phases of growth, GFP fluorescence was much lower in the *ipdC* mutant relative to WT (Fig. 7C). Western blot analyses determined that reduced GFP fluorescence was caused by reduced abundance of GFP in cells of the *ipdC* mutant regardless of phase of growth (Fig. 7D and E). We further showed that *gfp* transcript abundance was not affected in cells of the *ipdC* mutant compared to WT (Fig. 7F). These data suggest that the a decreased amount of GFP in the cells of the *ipdC* mutant strain may be the result of decreased translation and/or increased protein degradation.

**Fig 8:**
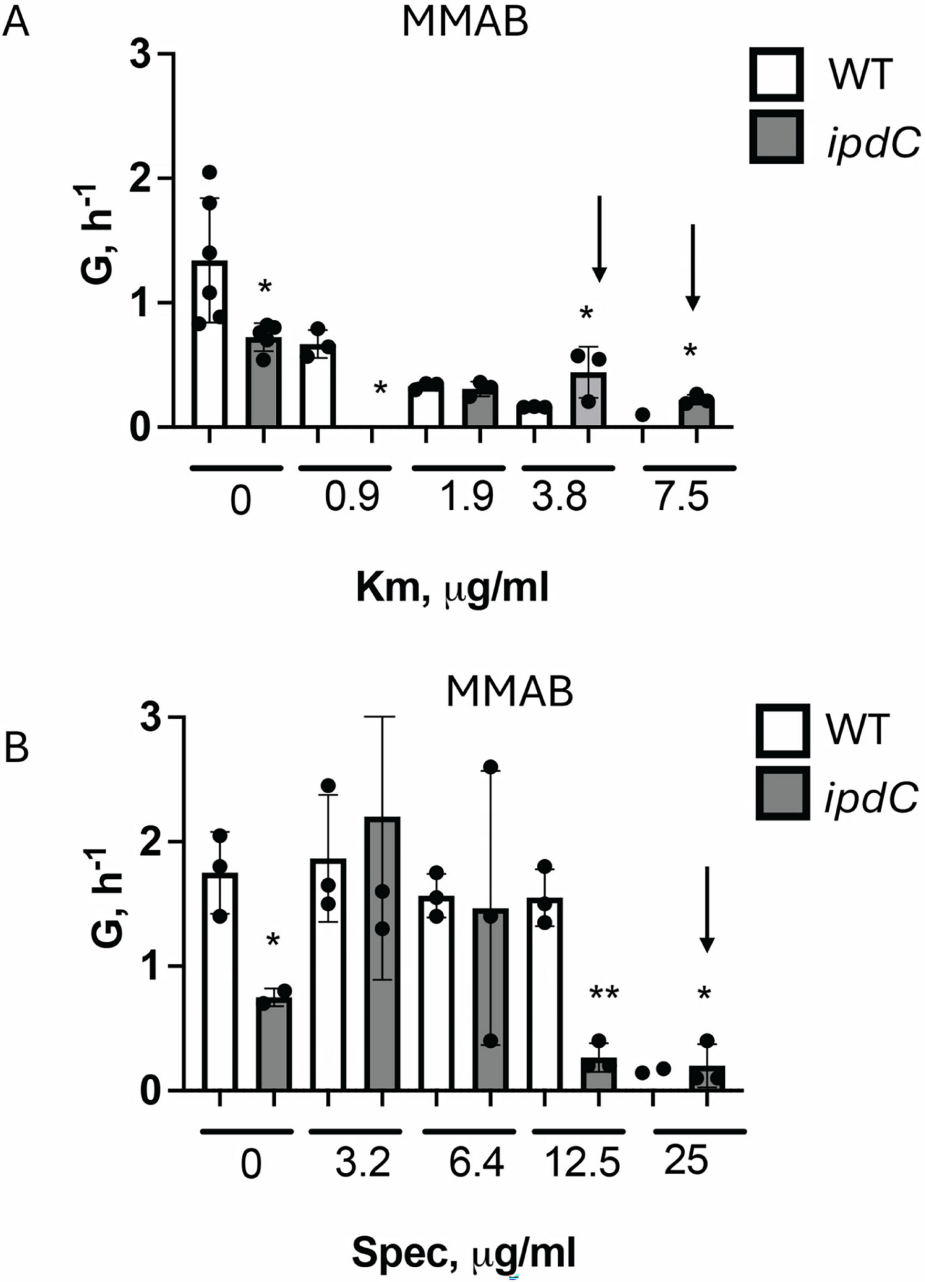
The *ipdC* mutant of *A. brasilense* has increased resistance to aminoglycoside antibiotics kanamycin and spectinomycin. Growth rates of WT and *ipdC* cells in MMAB supplemented with 0–7.5 µg/mL of kanamycin (**A**) or 0–25 μg/mL of streptomycin (**B**). Error bars represent the standard deviation (significantly different from control). *P < 0.1, **P < 0.01 (Student’s *t*-test).

To further test the activity of the translation machinery in the cells of the *ipdC* mutant strain, we treated these cells with sublethal concentrations of aminoglycoside antibiotics that interfere with ribosome activity, namely, kanamycin and spectinomycin, and measured cell growth rates (Fig. 8A and B). We found that the *ipdC* mutant was more resistant to sublethal concentrations of kanamycin (3.8 and 7.5 µg/mL) and spectinomycin (25 µg/mL) compared to the WT. The data suggest less active translation in the *ipdC* mutant strain compared to the WT.

### *ipdC* knockout and I3P interfere with *A. brasilense* attachment to wheat roots

**Fig 9:**
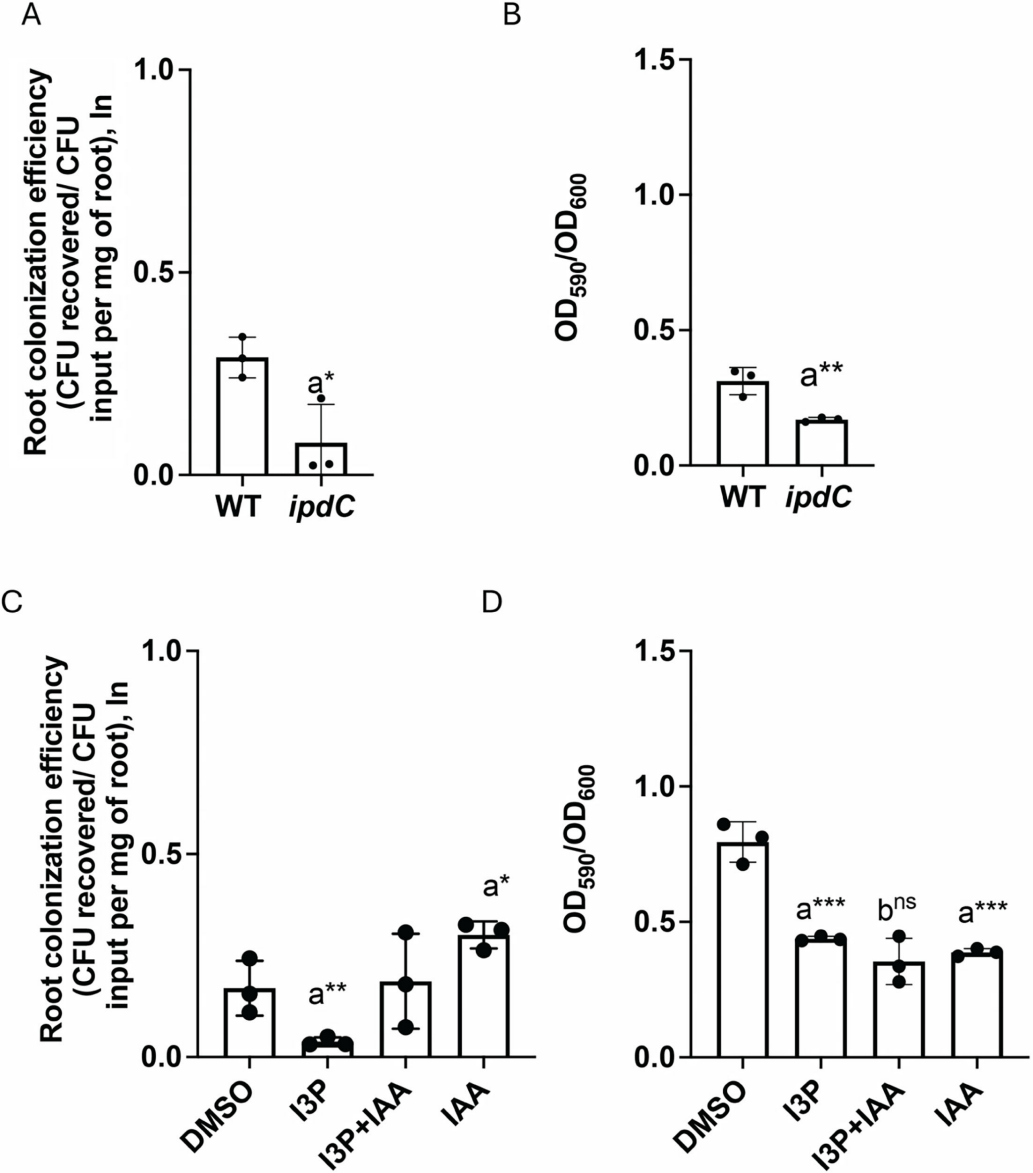
Wheat root colonization by WT and *ipdC* cells. (**A**) Root colonization efficiency by WT and *ipdC* cells. Error bars represent the standard deviation. a, significant in comparison with WT. *P = 0.1 (by Student’s *t*-test). (**B**) Biofilm formation by WT and *ipdC* cells. Error bars represent the standard deviation. a, significantly different from WT. **P = 0.01 (by Student’s *t*-test). (**C**) Root colonization efficiency by WT cells in the presence of 100 µM I3P, I3*P* + IAA, and IAA. DMSO was used as a control. Error bars represent the standard deviation. a, significantly different from DMSO. *P = 0.1, **P = 0.01 (by Student’s *t*-test). (**D**) Biofilm formation by WT in the presence of 100 µM I3P, I3*P* + IAA, and IAA. DMSO was used as a control. Error bars represent the standard deviation. a, significantly different from DMSO; b, not significantly different from I3P (ns). ***P = 0.01 (by Student’s *t*-test).

Given the role of indole derivatives in *A. brasilense* physiology, we next compared the ability of WT and *ipdC* mutant strains to colonize the roots of wheat (*Triticum aestivum*) seedlings germinated axenically (Fig. 9A). As expected from previously published findings by others (62–65), we found that the *ipdC* mutant strain colonized wheat roots significantly less in comparison with the WT strain (Fig. 9A). Biofilm formation was also affected in the *ipdC* mutant compared to the WT (Fig. 9B), which might explain lower root colonization. Next, we also tested how I3P and IAA added alone or in combination might affect the ability of the WT to colonize wheat root surfaces (Fig. 9C). While the addition of I3P decreased the ability of WT to colonize wheat roots, the addition of IAA increased the WT ability to colonize the root surfaces (Fig. 9C). We did not detect any differences in the plant root architecture or root weight with any of the added indole derivatives for the duration of the experiment (Fig. S2). This effect of IAA on *A. brasilense* wheat root colonization is consistent with data published by others (43, 49). Addition of IAA together with I3P returned the WT colonization of wheat roots to the levels of the control without indole derivative added (DMSO). These observations are consistent with IAA mitigating toxic effects of I3P on cells’ physiology. In contrast to root colonization, WT biofilm formation was affected in the presence of I3P and IAA added alone or in combination (Fig. 9D). The mitigating effects of IAA may thus be specific to bacterial physiology and plant-root association, but it is not evident in the colonization of abiotic surfaces. The mitigating effects of IAA in the presence of I3P on root surface colonization could perhaps be related to the positive effects of IAA on cell physiology within the wheat rhizosphere.

## DISCUSSION

Increasing evidence highlights IAA as a signal molecule in bacteria. IAA, as a secondary metabolite, influences gene expression related to cell division and growth, motility, enzyme production, biofilm formation, and the interaction with plant hosts (37, 39, 40, 66–68). IAA is thus not simply a by-product of bacterial metabolism but a bioactive compound mediating bacterial responses to environmental stresses and facilitating interactions with plant roots. In the present study, we provide evidence supporting the hypothesis that one of the physiological roles of IAA biosynthesis may be to protect *A. brasilense* from the toxic effect of indoles on membrane potential homeostasis. These findings imply that IAA produced by *A. brasilense* has a dual role in modulating interaction with plant roots but also in protecting bacterial energetics under conditions detrimental to membrane potential homeostasis.

Our results indicate that an *ipdC* mutant derivative has a reduced growth rate, an altered physiology, and a more depolarized membrane compared to the parent strain. The *ipdC* mutant is also impaired in wheat root surface colonization and biofilm formation, consistent with findings by others (62–64, 69). We also show that exogenous addition of indole precursors of IAA (I3P, TAM, and I3LA) also caused a reduction in the membrane potential of WT *A. brasilense*, at least at the concentrations we tested. Combined, these observations are consistent with an *A. brasilense ipdC* mutant accumulating indole derivatives that decreased membrane potential in the absence of IAA biosynthesis. These findings align with previous studies indicating that indole derivatives, although essential intermediates in IAA synthesis, can be toxic to bacterial cells (3, 61, 70). IAA accumulates during the prolonged stationary phase in *A. brasilense* and many other bacterial species (46, 66, 71, 72). IAA biosynthesis is also under the control of the stationary phase and stress resistance sigma factor RpoS in several bacteria, and treatment of cells with IAA has been shown to protect some soil bacteria from several abiotic stresses (48, 73). The exact mechanisms by which IAA protects against abiotic stresses are not known in most cases. Some of the protective effects may be related to the role of IAA in restoring a depolarized membrane potential identified here for *A. brasilense*.

Various stress factors affect protein synthesis, folding, and stability in bacteria (74). Our results show that conditions that lower the membrane potential of *A. brasilense* (mutation in *ipdC* or addition of indoles) also reduced protein translation. The reduced translation in the *ipdC* mutant was reported previously by others (51). In this study, the authors compared gene expression of *A. brasilense* WT and its *ipdC* mutant derivative in microarray experiments and found that genes coding for many ribosomal proteins were downregulated (51). The same study found that exogenous addition of IAA triggered the downregulation of several genes coding for ribosomal proteins, including the translation elongation factor Tu as well as of enzymes involved in oxidative metabolism and ATP synthesis (51). Our results here are fully consistent with the findings by these authors in showing reduced translation and membrane energetics homeostasis. The reduced translation is also consistent with observations made with persister cells, which have a reduced metabolism and membrane potential (27, 28), and with the effect of indoles in promoting ribosome hibernation (30). In *A. brasilense*, IAA is produced during bacterial growth, but it accumulates during prolonged stationary phase (72), which corresponds to nutrient stress conditions. IAA, other auxins, temperature, and nutrient stress, as well as suboptimal pH, also regulate the *ipdC* promoter activity and auxin production (54, 56). We find that one of the metabolite precursors of IAA, I3P, induces the *ipdC* promoter activity, and we confirm the effect of nutrient stress (stationary phase or lack of a reduced source of nitrogen) on *ipdC* promoter activity. We propose that a function of the IpdC enzyme-dependent pathway to IAA biosynthesis is to protect *A. brasilense’s* cellular physiology and membrane energetics under various stress conditions. IAA is an abundant molecule in the rhizosphere (66). IAA could also protect rhizosphere communities from abiotic stressors that likely abound, given the density of organisms and competition for resources in this environment. IAA was shown to be exchanged between members of cyanobacterial harmful algal bloom communities, where it triggers oxidative stress protection in at least some of the microbial members (75, 76). The protective effect of IAA against stressors could be a universal one.

Our findings indicate a protective role of IAA against the harmful effects of indole derivatives that negatively impact bacterial membrane potential. Two distinctive effects of IAA were detected: a short exposure to IAA could restore the membrane potential of the *ipdC* mutant but not that of WT cells whose membrane potential is depolarized by adding increasing concentrations of the I3P indole. We note that the discrepancy in the responses is not unexpected, given that the physiology of the *ipdC* mutant and that of WT cells exposed to increasing concentrations of I3P is not identical. The *ipdC* mutant lacks a functional IpdC enzyme and an associated IAA biosynthesis pathway that are present in the WT. Evidence here indicates reduced translation activity in the *ipdC* mutant compared to WT. The WT thus has the full complement of functions to adjust its physiology to the presence of I3P, including IpdC-mediated IAA biosynthesis to metabolize I3P and other indoles produced through this pathway or by adjusting the intracellular flux of indole metabolites to detoxify the cells. The greatest effect of IAA addition together with I3P on the WT cell’s membrane potential was under prolonged incubation (24 h). Addition of IAA at the concentration we selected for these experiments (100 µM) increased the membrane potential of WT cells at low concentrations of I3P and mitigated the toxic effects of I3P at elevated concentrations. A similar mitigation of IAA against the deleterious effects of I3P was observed for wheat root surface colonization, suggesting that the observations are relevant to the association with the plant. These data support the protective role of exogenous IAA on membrane potential and that the protective effect of IAA may be mediated through the adjustment of cells’ physiology. Our findings that I3P induces the *ipdC* promoter would imply that the protective effect of IAA is through increased activity of the IpdC pathway when the concentration of I3P increases.

In conclusion, results here indicate that a major signaling role of IAA and of the IAA biosynthesis pathway through IpdC in *A. brasilense* is maintenance of membrane potential homeostasis and regulation of translation. We surmise that during growth under optimal conditions, cells produce little IAA but have an active metabolism that turns over all intermediates quickly. Under limiting nutrient conditions or in the presence of stressors, growth slows along with metabolism, which results in the increasing accumulation of indoles, which could contribute to lowering the membrane potential further. Under these conditions, production of IAA or the ability to import and sense IAA within the environment to adjust cell physiology could provide bacterial cells with a survival advantage by mitigating some of the toxic effects of these indoles. IAA production is widespread in soil and rhizosphere bacteria, and the IpdC enzyme-pathway is predominant in many of these species (66). Thus, the role of IAA in *A. brasilense* physiology identified here may be conserved in diverse soil bacteria and could be significant in the rhizosphere.

## MATERIALS AND METHODS

### Bacteria strains, medium, and growth conditions

The strains and plasmids used in this study are listed in Table 1. *A. brasilense* strains were grown at 28°C with shaking, in the MMAB with malate as a carbon source and with or without ammonium chloride as the nitrogen source (77) or in the tryptone-yeast extract (TY) medium (yeast extract 5 g/L and tryptone 10 g/L) (78). The antibiotics were used at the following final concentrations: kanamycin, 30 µg/mL; tetracycline, 10 µg/mL; ampicillin, 200 µg/mL (for *A. brasilense*); and gentamycin, 20 µg/mL. *A. brasilense* Sp7 is naturally resistant to 200 µg/mL ampicillin (79). Indole derivatives I3P, IAA, TAM, I3A, and I3LA were used at final concentrations of 1, 25, 50, 75, 100, 150, 200, 300, and 450 µM from a 100 mM stock in anhydrous DMSO. Indole derivatives were added to bacterial cultures for 1 h (acute exposure) or 24 h (persistent exposure). Growth of the bacterial strains in the presence or absence of the indole derivatives was performed in sterile 96-well flat-bottom plates containing 180 µL of culture medium. OD_600_ measurements were taken every 20 min for 24 h using a BioTek ELx808 Ultra Microplate Reader. The growth rate of the cells was calculated using Gen5 software from BioTek. Growth experiments were performed with three to five biological replicates, with a minimum of three technical replicates each.

**TABLE 1:** Strains and plasmids used in this study^*a*^

Strain or plasmid	Description	Source
Strains		
Sp7	WT strain *Azospirillum brasilense*	ATCC 29145
*ipdC::Gm^r^*	*ipdC:: pKNOCK-Gm^r^* in *A. brasilense* Sp7 background, Gm^r^	This study
*E. coli* Top10	General cloning: F– *mcrA Δ*(*mrr-hsdRMS-mcrBC*) φ80*lacZDM15 ΔlacX74 recA1 araD139 Δ(ara leu)7697 galU galK rpsL* (Str^r^) *endA1 nupG*	Invitrogen
*E. coli* One Shot PIR1	F^–^ *∆lac* 169 *rpo*S(Am) *rob*A1 *cre*C510 *hsd*R514 *end*A *rec*A1 *uidA(∆Mlu* I)*::pir-116*	Thermo Fisher Scientific
*E. coli* HB101	Host strain for pRK2013	ATCC 33694
Sp7 (pBBR-MCS3)	*A. brasilense* Sp7 WT strain carrying pBBR-MCS3 (WT ev), Tet^r^	This study
*ipdC* (pBBR-MCS3)	*A. brasilense ipdC* mutant strain carrying pBBR-MCS3 (*ipdC* ev), Tet^r^	This study
*ipdC* (pBBR-*ipdC*)	*A. brasilense ipdC* mutant strain carrying pBBR-*ipdC* (*ipdC* IpdC^WT^), Tet^r^	This study
Sp7 (pFUS1)	*A. brasilense* Sp7 strain carrying pFUS1 empty promoter probe vector [WT(pFUS1)], Tet^r^	This study
Sp7 (pFUS-P*_ipdC_*)	*A. brasilense* Sp7 strain carrying pFUS- P*_ipdC_*, vector reporting on the ipDdC promoter activity [WT(pFUS- P*_ipdC_*)], Tet^r^	This study
Sp7 (pHRGFP)	*A. brasilense* Sp7 strain carrying pHRGFP [WT(pHRGFP)], Tet^r^	This study
*ipdC* (pHRGFP)	*A. brasilense ipdC* mutant strain carrying HRGFP [*ipdC*(pHRGFP)], Tet^r^	This study
Plasmids		
pRK2013	Mobilization helper plasmid, Rk2 replicon, Tra, and Km^r^	(80)
pKNOCK-Gm*^r^*	Vector for gene knock-in through Campbell insertion mutation	(81)
pKNOCK-*ipdC*	pKNOCK- Gm*^r^* vector with 200 bp of *ipdC* gene, Gm*^r^*	This study
pBBR1-MCS3	General cloning vector, Tet^r^	(82)
pBBR-*ipdC*	pBBR1-MCS3 vector with 600 bp of 5′ region from start site of ipdC and including the 1,638 bp region of *ipdC*, Tet^r^	This study
pFUS1	Broad-host-range vector with promoterless *gusA* Tet^r^	(83)
pFUS-P*_ipdC_*	pFUS1 vector containing 600 bp upstream of the start codon for *ipdC* (P*_ipdC_*), Tet^r^	This study
pHRGFP	pBBR1 origin plasmid expressing GFP, Tet^r^	(84)

^*a*^ ATCC, American Type Culture Collection; WT, wild type.

*A. brasilense* WT and *ipdC* mutant cells carrying a broad host range vector constitutively expressing GFP (pHRGFP) were constructed using triparental mating as described previously (84).

### *ipdC* insertion mutation and plasmid construction for ipdC complementation promoter activity

To construct an *ipdC* insertion mutant, a 200 bp sequence within the *ipdC* gene was cloned into the polylinker of the pKNOCK-Gm^r^ vector using XhoI-EcoRI restriction sites. The resulting pKNOCK-*ipdC* plasmid was transformed into *E. coli* One Shot PIR1 cells. The pKNOCK-*ipdC* plasmid was introduced into *A. brasilense* Sp7 by triparental mating using the pRK2013 vector as a helper, as described previously (83).

Primers used for *ipdC* insertional mutants were forward (5′-GGGTCTAGACTGGGGGCGAAGCGGACGGCG-3′) and reverse (5′-GCCAAGCTTGGCGTTGCCCTCCGTCGTG

CC-3′). The resulting strain (*ipdC::pKNOCK-Gm*^r^ (thereafter *ipdC*) was maintained with 20 µg/mL of gentamicin supplemented to the culture media. For complementation of *ipdC*, 600 bp of the upstream (promoter region) and the entire 1,638 bp of the *ipdC* open reading frame (ORF) were cloned into the XhoI-XbaI restriction site of the pBBR (Tet^r^) vector using forward (5′-TCGCTCGAGATCCCCTCCTCCTCACGCAAA-3′) and reverse (5′-ATTTCTAGATTATTCCCGGGGCGCGGCGTG-3′) primers. To create the P*_ipdC_* promoter reporter plasmid, 967 bp upstream of the *ipdC* ORF was cloned into the broad host range pFUS1 vector, at the 5′ end of the promoterless gusA gene, which encodes for β-glucuronidase (79).

### Colorimetric detection of extracellular and intracellular indole derivatives using Salkowski’s method

To measure the indole derivatives, we used Salkowski’s method as previously described for microplates (85) with the following modifications. Cell cultures were prepared in 100 mL flasks containing 25 mL each of MMAB, with malate as a carbon source and ammonium chloride as the nitrogen source with or without 150 µM L-tryptophan, added as an IAA precursor, with appropriate antibiotics and incubated at 30°C for 96 h. OD_600_ values were recorded for the strains. After incubation, cultures were centrifuged at 3,000 rpm for 10 min. The supernatants and samples were treated as described previously, except that after mixing with the Salkowski reagent in a separate tube (using a 500 µL supernatant-to-1 mL Salkowski reagent ratio), tubes were incubated 30 min at room temperature in the dark to let precipitating salts that interfere with the measurements settle at the bottom of the tubes, followed by collection of 125 µL of the supernatant for the microplate assay, as previously described (85). Uninoculated medium treated under identical conditions served as a blank. For the intracellular fraction, cell pellets were washed three times with ultrapure sterile water, re-suspended into 2 mL of ultrapure sterile water, and sonicated (15 s runs with 10 s pulses, over 12 cycles) to lyse the cells, followed by centrifugation at 3,500 rpm for 10 min to remove cell debris before removing a 125 µL aliquot of the supernatant. The amount of both extracellular and intracellular indole derivatives was estimated with a calibration curve by using IAA-known standard concentrations (10–100 μg/mL) and measuring absorbance of standards and samples at 530 nm, using a Spectramax plate reader. These experiments were performed with two biological replicates, with three technical replicates each.

### Microscopy

Bacterial cell membranes were stained as described previously (86). Briefly, 1 µL of 1 mg/mL FM4-64 (Biotium SynaptoredC2) was added to 100 µL of *A. brasilense* cultures grown to mid-exponential phase (OD_600_ = 0.6) or stationary phase (OD_600_ = 2.5) and incubated for 5 min at room temperature in the dark. Images were captured using a ×100 objective with oil immersion mounted onto a Nikon Eclipse 80i fluorescent microscope. Images of the cells stained with FM4-64 dye were collected using an argon ion laser with an emission/excitation at 515/630 nm. Cell length and width were measured using ImageJ Fiji (version 2.14.0/1.54f). Cell volume was calculated according to the formula *V* = [(*w*^2^ *π* / 4) × (*l* − *w*)] + (*π × w*^3^ / 6), where *l* is the length and *w* is the width of the cell. The staining technique employed here allows for differentiation between elongated and undivided cells and dividing cells. Experiments were performed three times, and combined data were used for the graph presented in the paper.

### β-Glucuronidase activity assay for promoter activity

Promoter activity of *ipdC* was determined by cloning promoter regions upstream of a promoterless *gusA* gene on the pFUS1 vector (Table 1) followed by quantitative evaluation of promoter activity using a high-throughput β-glucuronidase assay and *p*-nitrophenyl-β-d-glucuronide as a substrate essentially as described previously (87). Each culture was assayed in triplicate, in three independent experiments. Briefly, *A. brasilense* strains carrying an empty vector or with a vector with the cloned *ipdC* promoter region were grown overnight in rich medium (TY) supplemented with tetracycline, washed three times with 10 mM sterile phosphate buffer, and re-inoculated into MMAB without an organic nitrogen source (nitrogen-fixation conditions) or in MMAB with ammonium chloride as the nitrogen source. For nitrogen-fixation conditions, cultures were incubated at 28°C without shaking to limit aeration. Cultures containing ammonium chloride were incubated at 28°C with shaking for 15 h (OD_600_ = 0.6–0.8) and 24 h (OD_600_ = 1.2–1.5). Small volumes (125 µL) of the cultures were aliquoted into a 96-well flat-bottom plate. The plate was incubated in a −80°C freezer for 20 min followed by incubation on a heat block at 57°C for 20 min and cooling down on ice for 5 min. Then, 4 µL of *p*-nitrophenyl-β-D-glucuronide (ACROS Organics) at 17 mg/mL was added to each well at room temperature and mixed five times by pipetting. Reaction measurements were taken every 2 min for 1 h using an 800 TS absorbance reader with Gen5 software and a 405 nm filter (BioTek ELx808 Ultra Microplate Reader). The promoter activity of *ipdC* in the presence of tryptophan, indole-3-pyruvate, and IAA was done as described previously (88), with cells collected in the exponential (OD_600_ = 0.6–0.8) and stationary (OD_600_ = 1.2–1.5) phases of growth. Cell cultures were supplemented with 100 µM of IAA, L-tryptophan, or I3P to induce the *ipdC* promoter.

### Membrane potential measurement using DiSC_3_(5)

Measurement of membrane potential using DiSC3(5) was described previously (89). Briefly, *A. brasilense* strains were grown overnight at 28°C in the MMAB medium with antibiotics and washed with sterile 10 mM phosphate buffer (88). Cells were either re-inoculated (500 µL into 5 mL) into fresh MMAB and incubated 3 h at 28°C (to obtain exponentially grown cells) or re-inoculated into fresh MMAB and kept growing until the stationary phase. For membrane potential measurements, cultures were adjusted to an OD_600_ of 1.0, and 1 µL of 100 µM of DiSC3(5) was added to 100 µL of cells. Fluorescence was measured immediately at emission/excitation of 622/670 nm using the BioTek ELx808 Ultra Microplate Reader. CCCP (final concentration of 10 µM) was then added to the dye-bound cells, and the fluorescence was measured again as above. Measurements were taken using a BioTek ELx808 Ultra Microplate Reader every minute for 5 min before CCCP was added and every minute for 20 min after CCCP or DMSO was added. Experiments were performed using three to five biological replicates with a minimum of four technical replicates each. Representative data from one set of these experiments are shown.

### Western blotting

Whole-cell protein extracts of *A. brasilense* WT and *ipdC* strains harboring the pHRGFP plasmid (84) were isolated as described previously (90). Briefly, 25 mL of cultures were used for protein isolation using sonication with the following cycles: 30 s total with 5 s on and 10 s off cycle. For Western blot analysis, proteins were separated on a 10% SDS-PAGE gel and blotted onto a polyvinylidene difluoride membrane at 90 V for 1 h 10 min. The membrane was blocked with tris-buffered saline with 0.1% Tween 20 and 5% nonfat dry milk. YFP-tagged proteins were detected using an anti-GFP (91) antibody at a 1:2,000 dilution. Membranes were incubated with horseradish peroxidase-conjugated secondary antibodies (anti-rabbit, Abcam) at a 1:10,000 dilution and developed using the Bio-Rad imaging system. Protein abundance quantitation was performed using densitometry analysis in ImageJ Fiji.

### Sublethal antibiotic resistance assay

*A. brasilense* cells were cultured overnight in MMAB medium with antibiotics, washed with sterile 10 mM phosphate buffer, and diluted to OD_600_ = 0.1 in MMAB medium with ampicillin (WT strain) and gentamycin (*ipdC* strain) and kanamycin (0.4–7.5 µg/mL) or spectinomycin (3–25 μg/mL) and cultured using a microplate reader for 20 h. Growth rates were calculated using the Gen5 software (BioTek). I3P or IAA (100 µM) was added to the medium, together with kanamycin or spectinomycin, as needed.

### Plant root colonization assay

Wheat (*Triticum aestivum*) seedlings were used in the root colonization assay. Plant seeds were surface sterilized and germinated as described previously (92). The germinated seedlings were placed into 50 mL Falcon tubes containing 15 mL of semisolid (4 g/L noble agar) Fahraeus medium (92) and allowed to grow for two more days. All subsequent inoculations with WT and *ipdC* mutant strains were performed on 7-day-old germinated and surface-sterilized seedlings. For the root colonization assays, WT and *ipdC* mutant strains of *A. brasilense* were cultured in liquid MMAB medium with antibiotics overnight (28°C at 175 rpm). The cultures were washed three times with a sterile 10 mM phosphate buffer, normalized to an OD_600_ = 1.0, and further concentrated to a final OD_600_ = 2.5 with sterile 10 mM phosphate buffer. Twenty-five microliters of cells were injected into the medium about 15 mm away from the roots of the seedlings. All experiments were done in triplicate, with three biological samples used each time. Plants were grown for 5 days following bacterial inoculation. After incubation, the roots were washed three times with sterile phosphate buffer, followed by grinding in 400 µL of the same buffer. The ground roots’ supernatant was serially diluted and plated onto MMAB medium with appropriate antibiotics for CFU counts. Plates were scored after 48 h of incubation at 28°C. Root colonization efficiency was calculated as follows: ln [CFU (*t*5 roots) / CFU (*t*0) / g of wet roots], where *t*5 is the time at which the plants were sacrificed, 5 days post-inoculation, and *t*0 was the time of initial bacterial inoculation. To study the role of indole derivatives on wheat root colonization by the WT *A. brasilense* strain, the semisolid Fahraeus medium was supplemented with 100 µM of I3P, IAA, or I3*P* + IAA or an equal volume of DMSO used as a solvent for indole derivatives before the wheat seedlings were placed in the tube. All subsequent inoculations with the WT cell culture were done as described above. Experiments were performed in triplicate.

### Biofilm assay

Biofilm formation was determined using crystal violet staining. Briefly, cultures were grown overnight in MMAB, washed with sterile 10 mM sterile phosphate buffer, and adjusted to an OD_600_ of ~1.0. Next, 1 mL of cells was mixed with DMSO (control) or 100 µM of I3P or of IAA. Cells (180 µL) were aliquoted in wells of a 96-well plate and incubated at 28°C for 48 h without shaking. Next, biofilm formation was measured using 0.5% crystal violet solution. Plates were incubated for 30 min at room temperature and then carefully washed three times with tap water. Dye remaining on the surfaces of the microplate wells was extracted with 180 µL of 33% acetic acid. Next, the OD_590_ of the resulting solution in each well was determined using a microplate reader (BioTek ELx808 Ultra Microplate Reader). Data were normalized to total growth estimated by measuring OD_600_ of wells prior to crystal violet staining. Experiments were performed using three biological replicates with at least six technical replicates each.

### Statistical analyses

Statistical analyses were performed in GraphPad Prism (version 9). Two-tailed *t*-tests were applied for normally distributed data with 1 degree of freedom. Exact *n* values are provided in each figure legend.

## SUPPLEMENTAL MATERIAL

Supplemental figures aem.02384-24-s0001.pdf Figures S1 and S2.

ASM does not own the copyrights to Supplemental Material that may be linked to, or accessed through, an article. The authors have granted ASM a non-exclusive, world-wide license to publish the Supplemental Material files. Please contact the corresponding author directly for reuse.
