# Supplementary material for: Indole-3-acetic acid (IAA) protects Azospirillum brasilense from indole-induced stress
Source: Appl Environ Microbiol. 2025 Mar 25;91(4):e02384-24. doi: 10.1128/aem.02384-24 (PMC12016523; doi:10.1128/aem.02384-24)
Supplement: Supplemental figures — Figures S1 and S2. [file aem.02384-24-s0001.pdf]

Indole 3-acetic acid (IAA) protects *Azospirillum brasilense* from Indole-induced stress

Elena E. Ganusova[1], Ishita Banerjee[1], Trey Seats[1], and Gladys Alexandre[1]

[1] Biochemistry & Cellular and Molecular Biology Department University of Tennessee, Knoxville, TN

Supplemental material

Supplementary figure 1. Concentration of extracellular and intracellular indole derivatives produced by *A. brasilense* WT and *ipdC* mutant strains. Error bars represent the standard deviation. (n.s.- no significant difference between extracellular and intracellular indoles produced by WT and *ipdC* mutant strain with or without150 μM tryptophan [by one-way ANOVA]).

Supplementary figure 2. Fresh root weight of wheat plantlets inoculated with *A. brasilense* WT and the *ipdC* mutant strain, 7 days post inoculation (**A**) and in the presence of 100 μM indole derivatives (ID) in the medium (**B**). I3P: Indole-3-Pyruvic Acid; IAA: Indole-3-Acetic Acid. Error bars represent the standard deviation to the mean [by Student's t-test]).

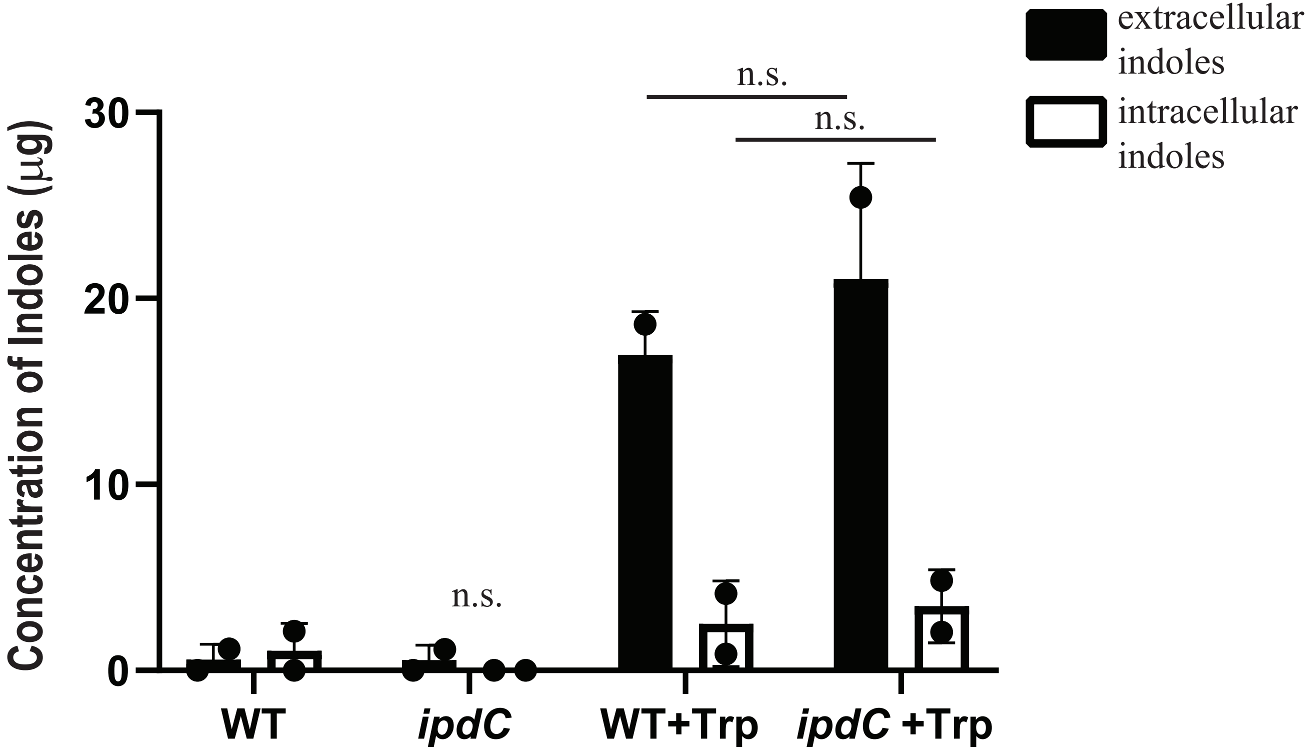

extracellular indoles
intracellular indoles
n.s.
n.s.
n.s.
Concentration of Indoles (μg)
30
20
10
0
WT
ipdC
WT+Trp
ipdC +Trp

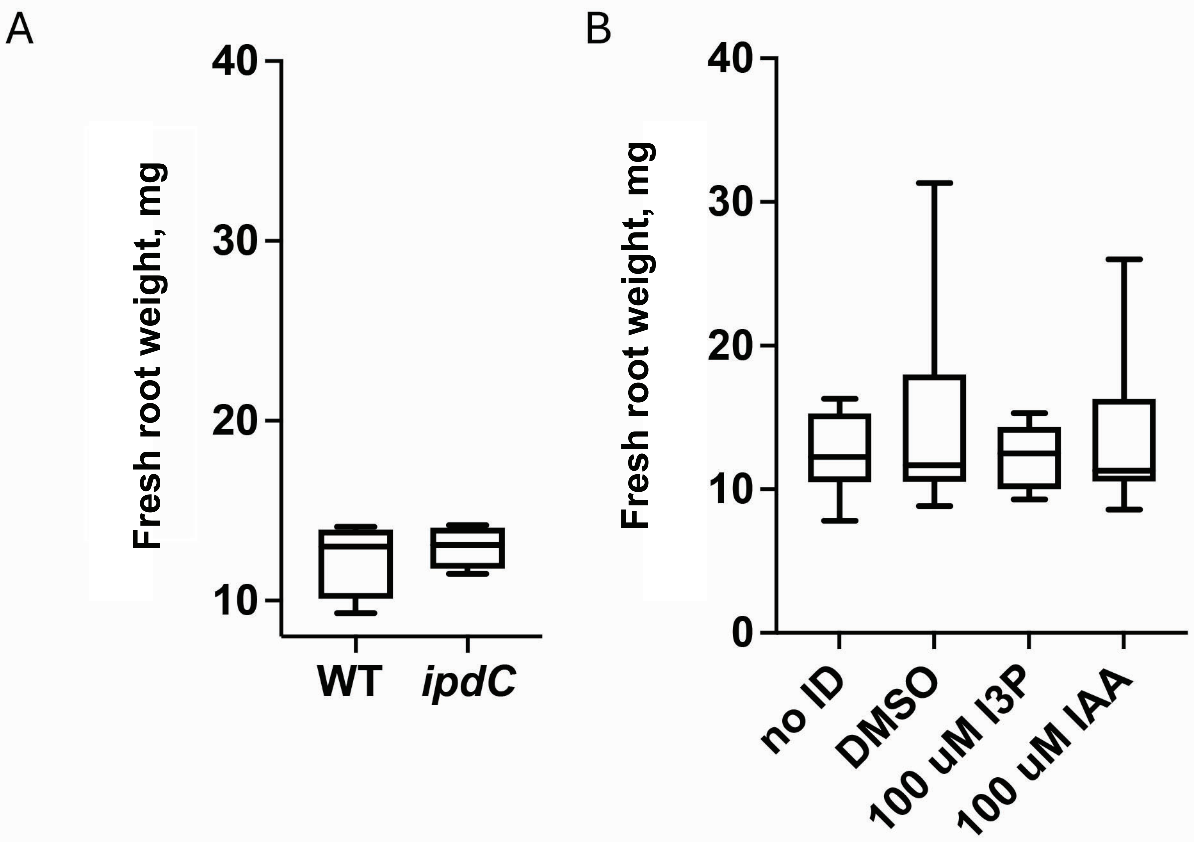
A
B
Fresh root weight, mg
40
30
20
10
WT
ipdC
Fresh root weight, mg
40
30
20
10
0
no ID
DMSO
100 uM I3P
100 uM IAA
